# Effects of the novel formin INF1 on ciliogenesis

**DOI:** 10.1186/2046-2530-4-S1-P33

**Published:** 2015-07-13

**Authors:** S Copeland, C Tenneson, W Kulacz, J Copeland

**Affiliations:** 1 Cellular and Molecular Medicine, University of Ottawa, Ottawa, Ontario, Canada

## Objective

INF1 is a novel member of the formin family of cytoskeletal regulatory proteins. We previously identified a connection between INF1 expression and microtubule acetylation and more recently have found that INF1 expression induces Golgi dispersion. Given the dependence of ciliogenesis on microtubule acetylation and Golgi-dependent trafficking we wished to determine the effects of INF1 expression on cilia formation.

## Methods

Transient transfection was used to express a series of INF1 derivative in NIH 3T3 cells and the effects on cilia formation, cilia length and Golgi dispersion were monitored by immunofluorescence. Anti-acetylated tubulin was used to visualize cilia and anti-Giantin was used to visualize Golgi morphology.

## Results

INF1 expression inhibited ciliogenesis in the majority of NIH 3T3 fibroblasts, however, inhibition of ciliogenesis was not connected to INF1-induced Golgi dispersion. A minority of INF1-expressing cells did form cilia and these were greatly elongated, the longest exceeding 70mm. A series of INF1 deletion derivatives were used to show that both inhibition of cilia formation and the induction of cilia elongation were dependent upon both the FH2 and microtubule-binding domains of INF1.

## Conclusion

The effects of INF1 expression on ciliogenesis were separate from its effects on Golgi-dispersion suggesting that Golgi untethering does not always inhibit ciliogenesis. The morphology of the elongated cilia formed in INF1 expressing cells is consistent with defects in dynein function.

